# Sensitizing non-small cell lung cancer to BCL-xL-targeted apoptosis

**DOI:** 10.1038/s41419-018-1040-9

**Published:** 2018-09-24

**Authors:** Qi Shen, Jun Li, Junhua Mai, Zhe Zhang, Andrew Fisher, Xiaoyan Wu, Zhaoqi Li, Maricela R. Ramirez, Shuqing Chen, Haifa Shen

**Affiliations:** 1 Department of Nanomedicine, Houston Methodist Research Institute, 6670 Bertner Avenue, Houston, TX 77030 USA; 2 Xiangya School of Medicine, Central South University, 410008 Changsha, Hunan China; 3 Department of Drug Metabolism and Drug Analysis, College of Pharmaceutical Sciences, Zhejiang University, 310058 Hangzhou, Zhejiang China; 4 Department of Cell and Developmental Biology, Weill Cornell Medical College, 1300 York Avenue, New York, NY 10065 USA; 5 Houston Methodist Cancer Center, Houston, TX 77030 USA

## Abstract

Lung cancer is the leading cause of death in the United States, with non-small cell lung cancers (NSCLC) accounting for 85% of all cases. By analyzing the expression profile of the pro-apoptotic and anti-apoptotic proteins, we have assigned NSCLCs into two distinct groups. While single agent treatment with the BCL-2/BCL-xL/BCL-w inhibitor ABT-263 (navitoclax) did not trigger apoptosis in either group, cells with a moderate to high level of MCL-1 expression were sensitive to ABT-263 treatment when MCL-1 expression was suppressed with a gene-specific siRNA. In contrast, those with a low MCL-1 expression did not undergo apoptosis upon combination treatment with ABT-263 and MCL-1 siRNA. Further studies revealed that cells with a low MCL-1 expression had low mitochondrial priming, and treatment with the chemotherapy drug docetaxel raised the mitochondrial priming level and consequently sensitized cells to ABT-263. These results establish a rationale for molecular profiling and a therapeutic strategy to treat NSCLC patients with pro-apoptotic anti-cancer drugs based on their MCL-1 expression level.

## Introduction

Lung cancer is the leading cause of cancer death among all cancer types. Therefore, breakthroughs in lung cancer treatment have the potential to save tens of thousands of patients every year.

The BCL-2 family of proteins play an essential role in mediating cell apoptosis as a means for the body to remove aging and abnormal cells. Members of the BCL-2 family contain one or more BCL-2 homology (BH) domains and can be divided into three subgroups based on their structure and function: the anti-apoptotic proteins (e.g., BCL-2, BCL-xL, BCL-w, MCL-1, and BFL-1), the multi-BH domain effector proteins (e.g., BAK, BAX, and BOK), and the pro-apoptotic BH3-only proteins. The pro-apoptotic BH3-only proteins can be further separated into direct activators (e.g., BIM, BID, and PUMA) and sensitizers (e.g., BAD, BIK, BMF, HRK, and NOXA)^1,2^. Activation of effector proteins leads to permeabilization of the mitochondrial outer membrane, which triggers apoptosis through the release of cytochrome C and subsequent activation of caspases. The anti-apoptotic proteins prevent the activation of effector proteins either through direct interaction or by inhibiting pro-apoptotic BH3-only proteins. Based on the same concept, small molecule inhibitors targeting the anti-apoptotic proteins (BH3 mimetics) have been developed to promote cancer cell apoptosis^3^. Certain inhibitors only target one specific member of the anti-apoptotic proteins, such as the BCL-2-specific inhibitor venetoclax (ABT-199)^4^, while others impact multiple proteins, as in the case of the BCL-2/BCL-xL/BCL-w inhibitor navitoclax (ABT-263)^5^. The BCL-2 family protein-targeted therapy is efficacious in treating hematopoietic malignancies^6,7^. However it has been reported that only a small fraction of NSCLC cells and breast cancer cells respond well to navitoclax treatment^8,9^, suggesting additional factors may play important roles in cell survival in these tumor types. Indeed, it has been shown that MCL-1 is another key pro-survival factor in NSCLC and breast cancer^10,11^.

In this study, we examined the response to treatments targeting the anti-apoptotic proteins in NSCLC. Our results indicate that the BH3 mimetic drugs can be applied to treat NSCLC patients and that the treatment strategy should be customized based on the gene expression profile of the tumor.

## Materials and methods

### Cell lines and cell culture

MRC-5, H460, H1299, H358, A-427, SW900, A549, H441, SK-LU-1, Calu-6, and H727 cells were obtained from ATCC (2012-2017). All cells were expanded and stored in liquid nitrogen when received and original vials were thawed for the experiments. No further authentication was performed. MRC-5, SK-LU-1, and Calu-6 were maintained in Eagle’s minimal essential medium (EMEM, HyClone, Logan, Utah, USA) supplemented with 10% fetal bovine serum, and the other cell lines were grown in the RPMI1640 medium (HyClone, Logan, Utah, USA) supplemented with 10% fetal bovine serum and 2 mM glutamine. Cell cultures were kept in 37 °C incubators with 5% CO_2_. All cells were verified mycoplasma-free using the MycoAlert™ Mycoplasma Detection Kit (#LT07-418, Lonza, Rockland, ME, USA) and were passaged for less than 6 months after resuscitation.

### siRNA transfection and Western blot analysis

All siRNA oligos were purchased from Sigma-Aldrich (Woodlands, Texas, USA). Their sequences are listed in Table 1. Cells were transfected using lipofectamine RNAiMax (#13778150, Life Technologies, Carlsbad, CA, USA) as the transfection reagent following the manufacturer’s instructions. Cells were lysed on ice for 20 min with the radioimmunoprecipitation assay (RIPA) buffer containing protease and phosphatase inhibitors, and cell lysate was then centrifuged at 14,000 rpm for 10 min at 4 °C. The protein concentration in the supernatant was measured using a bicinchoninic acid (BCA) protein assay kit, and the protein levels were analyzed with SDS-PAGE and Western blotting using an ECL kit (#34076, Thermo scientific, Waltham, MA, USA) for detection. Antibodies against the following proteins were used in Western blot analysis: BAX (#5023, Cell Signaling, Beverly, MA, USA), BAK (#6947, Cell Signaling, Beverly, MA, USA), BIM (#2933, Cell Signaling, Beverly, MA, USA), MCL-1 (#5453, Cell Signaling, Beverly, MA, USA), BCL-2 (#2872, Cell Signaling, Beverly, MA, USA), BCL-xL (#2762, Cell Signaling, Beverly, MA, USA), PUMA (#4976, Cell Signaling, Beverly, MA, USA), and β-Actin (#A2228, Sigma, St Louis, MO, USA).

**Table 1:** siRNA oligos used in this study

siRNA	Sequence
siMCL-1a	5′-CAUGCUUCGGAAACUGGACAUCAAA-3′
siMCL-1b	5′-GCAGUCCUCUAGUGUUUCA-3′
siBIM	5′-GACCGAGAAGGUAGACAAU-3′
siBID	5′-GAAGACAUCAUCCGGAAUA-3′
siBAX	5′-GCUCUGAGCAGAUCAUGAA-3′
siBCL-xL	5′-GGUAUUGGUGAGUCGGAU-3′
siBAK	5′-GUACGAAGAUUCUUCAAAU-3′
siSCR	5′-CCUCUUGAUGAACCAUCUA-3′

### Apoptosis assay

Cell apoptosis was measured using an Annexin V-PE kit (#88-8102-74, eBioscience, San Diego, CA, USA) following the manufacturer’s protocol. Sytox blue (#S34857, Life Technologies, Carlsbad, CA, USA) was used to replace 7-AAD in the assay. Apoptotic cells were detected with a BD LSRII flow cytometer and the results were analyzed using the BD FACSDiVa software.

### BH3 profiling

Plate-based BH3 profiling was performed in whole cells as described^12^. Briefly, digested cells were suspended in DTEB buffer (135 mM trehalose, 50 mM KCL, 20 μM EDTA, 20 μM EGTA, 0.1% BSA, 5 mM succinate, 10 mM HEPES-KOH pH 7.5) at a density of 2.5 × 10^6^ cells/mL, and the cell suspension was incubated for 10 min with an equal volume of the 4 × Dig/JC-1 Mix (4 μM JC-1, 40 μg/mL oligomycin, 20 mM 2-mercaptoethanol, and 0.01% digitonin [w/v] in DTEB) to produce a 2 × Dig/JC-1/Cell Mix. 15 μL of the 2 × Dig/JC-1/Cell Mix was added into each well containing 15 μL of either the 2 × peptide solution (120 or 240 μM) or the positive control carbonyl cyanide-4-(trifluoromethoxy)-phenyl hydrozone (FCCP, 10 μM). The plate was kept in dark and incubated at 20 °C with gentle shaking for 3.5 h. Fluorescent intensity was measured with a Synergy H4 plate reader (BioTek, Winooski, VT, USA) using an excitation wavelength of 545 nm and an emission wavelength of 590 nm. The percentage of depolarization for each peptide was calculated as: Depolarization% = 1 − (Sample-FCCP)/(DMSO-FCCP), where “DMSO” and “FCCP” refer to fluorescent intensities of the negative and positive controls respectively, and “Sample” refers to the fluorescent intensity of the test peptide. The JC-1 and FCCP were purchased from Santa Cruz Biotechnology (Santa Cruz, CA, USA). The PUMA, BAD, NOXA, and PUMA2A peptides were purchased from Selleckchem (Houston, TX, USA), and their sequences were the same as reported^12^.

### CRISPR/Cas9 knockout of MCL-1 or PUMA in cells

Calu-6 and A549 cells were transfected with the MCL-1 CRISPR/Cas9 KO Plasmid (#sc-400079, Santa Cruz Biotechnology, Santa Cruz, CA, USA) and PUMA CRISPR/Cas9 KO Plasmid (#sc-400464-2, Santa Cruz Biotechnology, Santa Cruz, CA, USA) using lipofectamine 3000 (#L3000008, Life Technologies, Carlsbad, CA, USA) as the transfection reagent, respectively. GFP-positive cells were collected 2 days after transfection by cell sorting using a BD FACSAria II cell sorter and seeded in 6-well plates to allow colonies to form. MCL-1 or PUMA expression in single cell clones was analyzed by Western blot analysis.

### MCL-1 overexpression in H727 or A549 cells

H727 or A549 cells were transfected with a MCL-1 overexpression plasmid (#SC315538, Origene, Rockville, MD, USA) using lipofectamine 3000 as the transfection reagent. The MCL-1 expression level was detected 2 days after transfection. Cells transfected with the vector PCMV6-XL4 (#PCMV6XL4, Origene, Rockville, MD, USA) served as a negative control.

### Cell viability assay

Cell viability was assessed with the 3-(4,5-dimethylthiazol-2-yl)-2,5-diphenyltetrazolium bromide (MTT, #M6494, Life technology, Carlsbad, CA, USA) assay following the manufacturer’s instructions. Briefly, A549 and H727 cells were seeded at 2,500 and 5,000 cells per well in a 96-well plate, respectively. They were treated with either chemotherapy drugs, ABT-263, or their combination the next day. After 72 h of incubation, 10 μL of MTT solution (5 mg/mL) was added to each well. The cell suspension was removed 4 h later, and formazan crystals that had formed in live cells were dissolved in DMSO. Absorbance was then measured with a 96-well plate reader.

### Efficacy evaluation in orthotopic lung cancer models

All animal studies were performed following protocols approved by the Institutional Animal Care and Use Committee (IACUC) at Houston Methodist Research Institute (animal protocol #0317-0009). Female athymic nude mice (strain code #490, 4–5 weeks) were obtained from Charles River (Wilmington, MA, US). Calu-6, Calu-6 KO, and A549 cells were transfected with a luciferase-expressing lentivirus as previously described^13^. Cells were mixed in a PBS/Matrigel suspension, and 2 × 10^5^ cells were inoculated into each mouse by intra-lung injection. 4 days after injection, mice with detected tumor growth were treated every other day with ABT-263 (80 mg/kg, MedChem Express, Princeton, NJ, USA) alone or in combination with a weekly dose of docetaxel (4 mg/kg, Accord Healthcare, Durham, NC, USA). ABT-263 was formulated in ethanol/ PEG 400/ Phosal 50 PG (10/30/60, vol/vol). ABT-263 was administered by gavage and docetaxel was injected intraperitoneally. Tumor growth was monitored using a Xenogen IVIS-200 imaging system (Caliper Life Sciences, Hopkinton, MA, USA) 10 min after an intraperitoneal injection of 150 mg/kg D-luciferin sodium salt (Gold Bio Technology, St. Louis, MO, USA). For the terminal deoxynucleotidyl transferase mediated dUTP nick end labeling (TUNEL) assay, mice bearing Calu-6, Calu-6 KO, and A549 tumors started treatment 35, 70, and 35 days after tumor injection, respectively. Tumor samples were collected 24 h after the second treatment of ABT-263, and tissue blocks were stained with a TUNEL assay kit (Roche Diagnostics, Indianapolis, IN, USA) to detect apoptotic cells. The percentage of TUNEL positive cells was calculated as the ratio of TUNEL positive nuclei to the total amount of nuclei in five random fields at 400× magnification.

### Analysis of mutation and gene expression in human NSCLC using TCGA data

Gene mutations and patient survival data from 230 lung adenocarcinoma samples^14^ were downloaded from the TCGA database using cBioPortal^15,16^. These data were used to calculate the percentage of gene alterations. Eight patients with 0 month survival between initial diagnosis and death were excluded from the survival analysis. Patient survival was displayed in a Kaplan-Meier plot, and a Gehan-Breslow-Wilcoxon test was applied to determine the statistical significance between survival curves using the Graphpad Prism v5.01 software.

### Microarray analysis

NSCLC tissue microarrays were purchased from US Biomax Inc (#BC04022, Rockville, MD, USA). Briefly, the microarray slides were deparaffinized using xylene, rehydrated in a descending series of ethanol and water, then boiled in 10 mM citrate buffer for 10 min. Endogenous peroxidase was applied in 3% hydrogen peroxide in water for 10 min followed by blocking with 2.5% horse serum for 20 min. Primary antibodies against MCL-1 (1:25, #MABC43, EMD Millipore, Billerica, MA, USA) and BCL-xL (1:300, #2764, Cell Signaling, Beverly, MA, USA) were incubated for 1 h at room temperature. The microarray slides were then washed twice for 5 min in TBST and incubated for 30 min with ImmPRESS HRP Anti-Mouse IgG (Ready to use, #MP-7402, Vector Laboratories, Burlingame, CA, USA) or Anti-Rabbit IgG (Ready to use, #MP-7401, Vector Laboratories Burlingame, CA, USA) secondary antibodies against the appropriate species. After washing twice for 5 minutes in TBST, bound antibodies were detected using the Liquid DAB + Substrate Chromogen System (#K3468, Dako, Carpinteria, CA, USA). The microarray slides were counterstained with hematoxylin, dehydrated with ethanol, cleared with Xylene, and then mounted with Cytoseal-60. Images were taken using a Nikon Eclipse 80i microscope with the same magnification.

### Determination of synergy

The interaction between docetaxel and ABT-263 was characterized using a constant drug ratio, and CompuSyn, Inc. software program was used to determine the combination index (CI)^17^. Based on the manufacturer’s instructions, the classification of drug interaction was used as follows: a CI of 1.2–1.45 indicates moderate antagonism, a CI of 0.85–1.2 indicates additive effect, a CI of 0.7–0.85 indicates moderate synergism, a CI of 0.3–0.7 indicates synergism, a CI of 0.1–0.3 indicates strong synergism, and a CI of <0.1 indicates very strong synergism.

### Statistical analysis

The Gehan-Breslow-Wilcoxon test, one-way ANOVA test, and one-tailed Spearman’s correlation test were applied to perform statistical analysis using the GraphPad Prism software v5.01. Results are presented as mean ± SEM. Statistical significance was defined as *p < 0.05 and **p < 0.01.

## Results

### BCL-2 family proteins are up-regulated in NSCLC

**Fig. 1:**
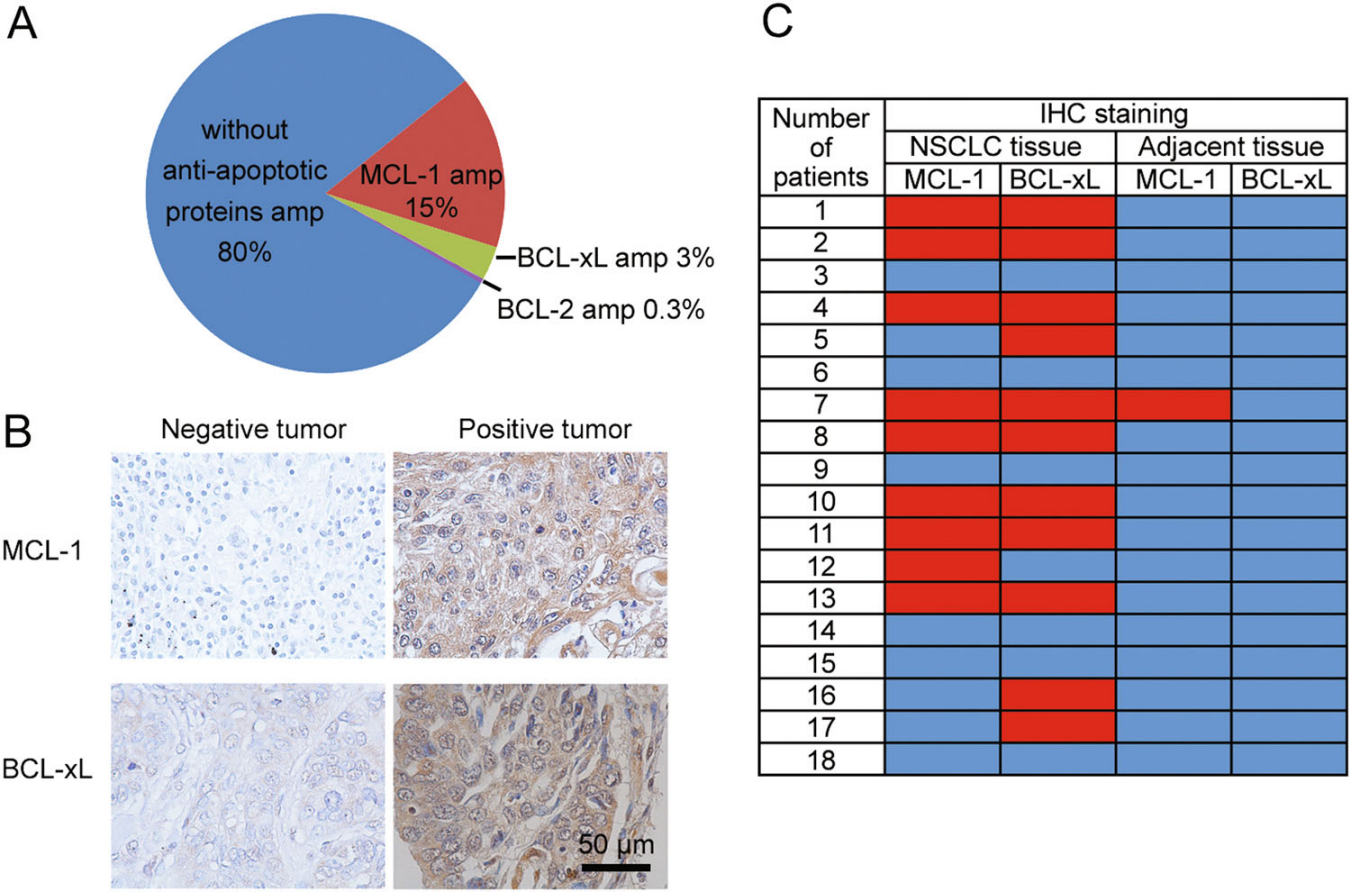
MCL-1 and BCL-xL are upregulated in a subset of NSCLCs. **a** Analysis of TCGA database on MCL-1 and BCL- xL gene amplifications in NSCLC patients (*n* = 230). amp, amplification. **b** Representative immunohistochemical staining results of NSCLC tissue. **c** Summary of immunohistochemical staining results of NSCLC tissue and adjacent normal lung tissue. Red indicates positive immunohistochemical staining, and blue indicates negative immunohistochemical staining

TCGA data analysis revealed around 15 and 3% of lung adenocarcinoma patients had an amplification in the MCL-1 gene and BCL-xL gene, respectively (Fig. 1a), although no correlation between their amplification and the overall survival was established in these patients (Fig. S1A, B). Amplification of BCL-2 was only detected in one sample from 230 patients (Fig. 1a). The result correlates with previous findings that NSCLC cells express a low level of BCL-2 and high levels of BCL-xL and MCL-1 proteins^10^. After validating the specificity of the antibodies (Fig. S1C), we also surveyed the expression profiles in 18 primary NSCLC tissues as well as their adjacent normal lung tissues via immunostaining. MCL-1 and BCL-xL expression were detected in 50% and 61% of the tumor samples, respectively (Fig. 1b, c). Most tumor samples with high MCL-1 levels also expressed BCL-xL, while neither MCL-1 nor BCL-xL was detected in one-third of the 18 samples (Fig. 1c). In contrast, only one out of 18 adjacent normal lung tissue samples showed a high expression of MCL-1, and BCL-xL was undetectable in all normal specimens (Fig. 1c and Fig. S1D). It has been previously demonstrated that the BCL-2 family proteins are dynamically regulated during the transcriptional, post-transcriptional, and post-translational processes^18–20^. The multiple levels of regulation most likely contributed to the much higher MCL-1 and BCL-xL-positive tissue microarray samples than in the TCGA database. Overall, these results indicate the up-regulation of the BCL-2 family proteins in NSCLCs.

### Suppression of MCL-1 expression sensitizes a subpopulation of NSCLC cells to ABT-263

**Fig. 2:**
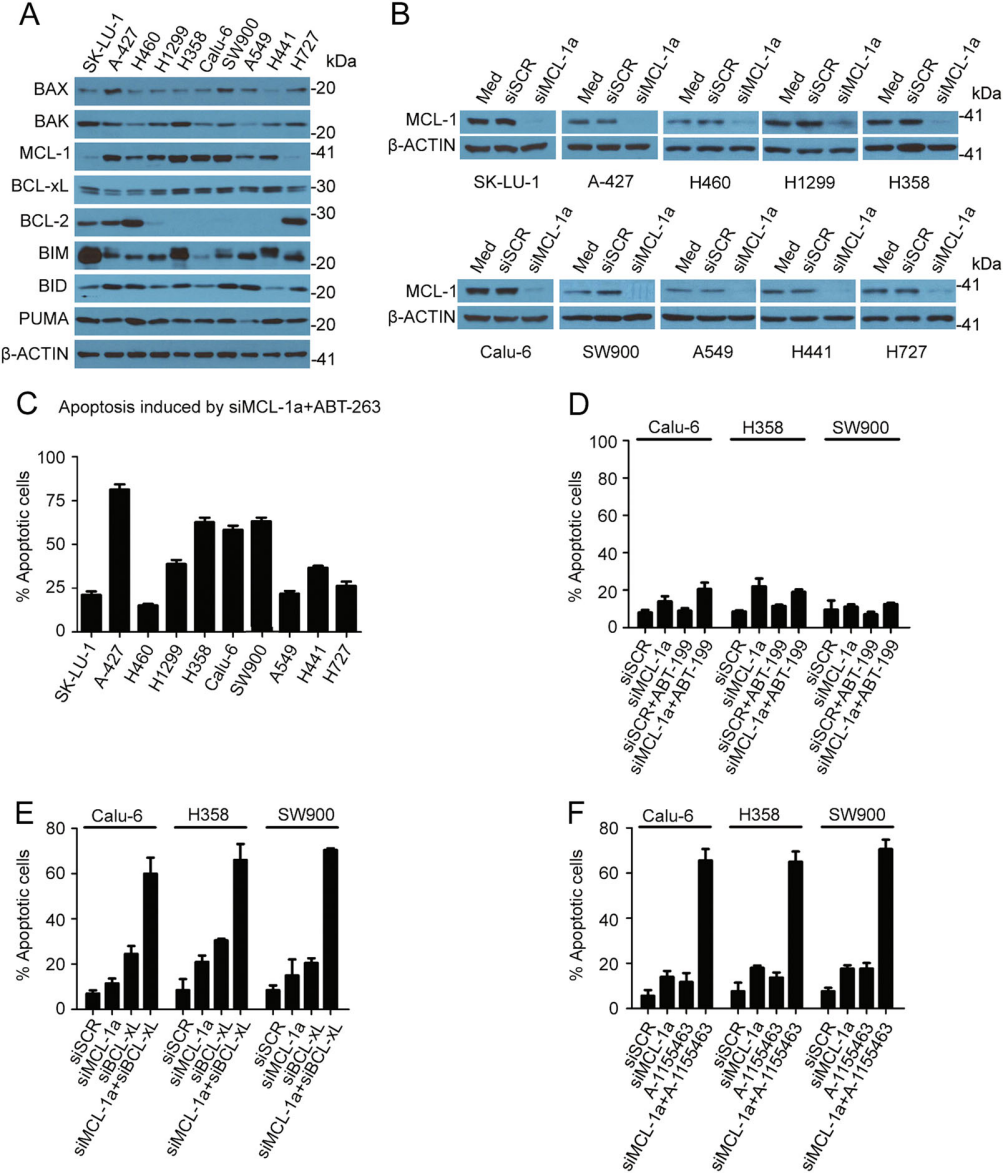
Variable apoptotic response from NSCLS cells to MCL-1 and BCL-xL inhibition. **a** Expression profile of BCL-2 family proteins in NSCLC cells. **b** Knockdown of MCL-1 expression in NSCLC cells 48 h after transfection with 25 nM siRNA oligos. siSCR: scramble siRNA; siMCL-1a: MCL-1a siRNA. **c** The percentage of apoptotic cells after siMCL-1a and AT-263 treatment. Cells were transfected with 25 nM siMCL-1a for 48 h and then treated with 1 μM ABT-263 for 36 h. Cell apoptosis was measured with flow cytometry. There were 3 samples in each group. **d** The percentage of apoptotic cells after siMCL-1a and ABT-199 treatment. Cells were transfected with 25 nM siMCL-1a or siSCR for 48 h and then treated with 1 μM ABT-199 for 36 h. Cell apoptosis was measured with flow cytometry. There were three samples in each group. **e** The percentage of apoptotic cells after siMCL-1a and siBCL-xL treatment. Cells were transfected with 25 nM siRNA oligos for 72 h and then analyzed for cell apoptosis with flow cytometry. There were three samples in each group. **f** The percentage of apoptotic cells after siMCL-1a and A-1155463 treatment. Cells were transfected with 25 nM siRNA oligos for 48 h then treated with 100 nM A-1155463 for 36 h. Cell apoptosis was measured with flow cytometry. There were three samples in each group

Previous studies have revealed critical roles of BCL-xL and MCL-1 in tumor cell response to BCL-2/BCL-xL/BCL-w inhibition^11,21^. We took a panel of ten NSCLC cell lines to examine their sensitivity to the BCL-2/BCL-xL/BCL-w inhibitor ABT-263. Western blot analysis showed that all cells had detectable levels of BCL-2 family proteins except for BCL-2 which was only expressed in 50% of cell lines (Fig. 2a), and levels of MCL-1, BIM, and BID varied dramatically among the cell lines. Consistent with a previous report^8^, treatment with 1 μM ABT-263 for 36 h did not trigger massive apoptosis in NSCLC cells (Fig. S2A, comparing the 1st and 3rd columns). To examine whether the MCL-1 protein level was critical for cell survival, we knocked down MCL-1 expression in these cell lines (Fig. 2b). While knockdown of MCL-1 alone did not induce cell apoptosis (Fig. S2A, 2nd column), co-treatment with ABT-263 caused a large amount of cell death (over 56% apoptotic cells) in A-427, H358, Calu-6, and SW900 cells, and moderate cell death (35–40% apoptotic cells) in H441 and H1299 cells (Fig. 2c and Fig. S2A). A cell viability assay also showed that, after MCL-1 knockdown, the sensitive cell lines (Calu-6, H358, and SW900) had significantly lower IC50 values for ABT-263 than the resistant cell lines (H460, A549, and H727) (Fig. S2B, C). On-target inhibition of BCL-2/BCL-xL/BCL-w and MCL-1 was confirmed when H358 cells were treated with a second siMCL-1 (siMCL-1b) and another BCL-2/BCL-xL/BCL-w inhibitor (ABT-737), which resulted in over 60% cell apoptosis (Fig. S2D and E). We further confirmed that cell death was a result of direct inhibition of BCL-xL and MCL-1. Co-treatment with the BCL-2-specific inhibitor ABT-199 and siMCL-1 did not trigger significant apoptosis in Calu-6, H358, or SW900 (Fig. 2d), while siMCL-1 combined with either the BCL-xL-specific inhibitor A-1155463 or knockdown of BCL-xL resulted in comparable levels of apoptosis in these cells (Fig. 2e, f and Fig. S2F). Cell apoptosis from the combination treatment was specific for the cancer cells, as treatment of the MCR-5 normal lung cells with siMCL-1a and ABT-263 did not induce cell death (Fig. S3A, B).

Recently, it has been reported that S63845 was a potent and selective small-molecule inhibitor of MCL-1. We treated NSCLC cells with S63845, ABT-263, or their combination, and measured cell apoptosis. A-427 was the only cell line to show a moderate level of cell death (around 39% apoptotic cells) after a single treatment of S63845, and heterogeneous apoptotic responses were observed among the 10 NSCLC cell lines when S63845 was used in combination with ABT-263 (Fig. S3C). There was a correlation on the induction of cell apoptosis between siMCL-1 and S63845 in the combination studies with ABT-263 (Fig. S3D), proving that S63845 specifically inhibited MCL-1 in these NSCLC cells.

### MCL-1 expression level correlates with the sensitivity to BCL-xL/MCL-1 inhibition

**Fig. 3:**
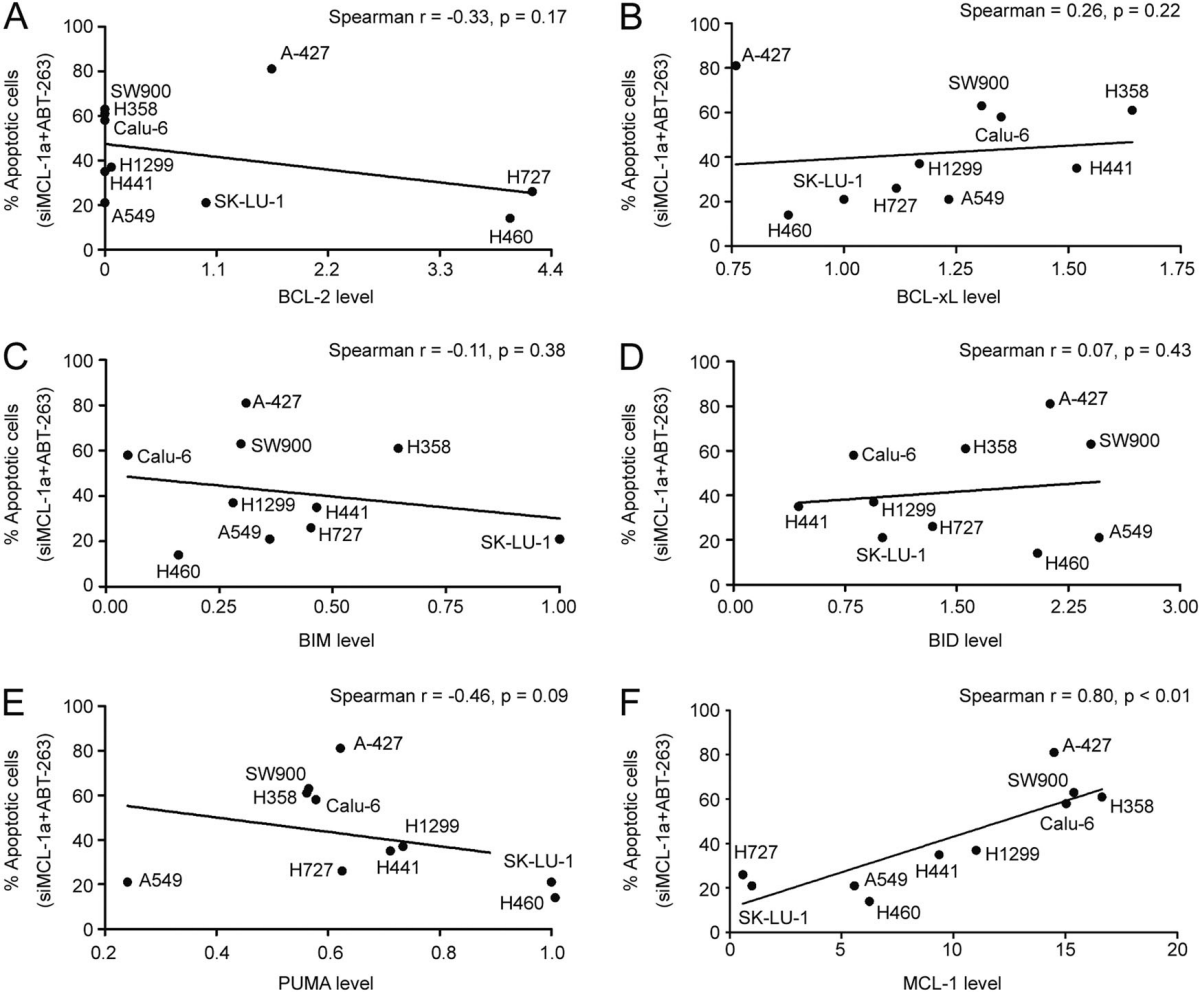
MCL-1 expression level correlates with sensitivity of NSCLC to MCL-1 and BCL-xL inhibition. Expression levels of BCL-2, BCL-xL, BIM, BID, PUMA, and MCL-1 were quantified using the Image Studio Ver 5.0 software. The level for each protein in SK-LU-1 cells was set as 1, and relative expression levels in other cells was calculated by comparing to SK-LU-1 cells. **a**–**f** The percentages of apoptotic cells induced by knockdown of MCL-1 combined with ABT-263 were plotted against relative expression levels of individual proteins in the NSCLC cells. The non-parametric one-tailed Spearman’s correlation test was applied to perform statistical analysis

Our analysis has shown that MCL-1 plays an important role in mediating intrinsic resistance to BCL-xL inhibition in a subset of NSCLC cell lines. A correlation study between the expression levels of BCL-2 family members and the apoptotic response to ABT-263 and siMCL-1 combination treatment indicated that BCL-2 and BCL-xL were poor predictors in NSCLC (Fig. 3a, b). Previous studies have demonstrated that BIM and BID are key mediators of apoptosis in cancer^22,23^. However, neither BIM nor BID expression could predict apoptotic response to combined suppression of MCL-1 and BCL-xL (Fig. 3c, d). A recent study suggested that PUMA might be another essential direct activator in mediating programmed cell death^24^, yet the PUMA expression did not correlate with the apoptotic response either (Fig. 3e). Our result demonstrated that MCL-1 expression was the only factor that significantly correlated with the percentage of apoptotic cells caused by ABT-263 and siMCL-1 combination treatment (Fig. 3f). Interestingly, cells can be divided into two distinctive groups. The six cell lines with a moderate level of MCL-1 expression (A-427, H358, Calu-6, H441, H1299, and SW900) responded positively to ABT-263 and siMCL-1 treatment, while the other four lines with a low level of MCL-1 expression (SK-LU-1, A549, H460, and H727) did not undergo apoptosis upon combination treatment.

### Cells resistant to BCL-xL/MCL-1 inhibition have low mitochondrial priming

**Fig. 4:**
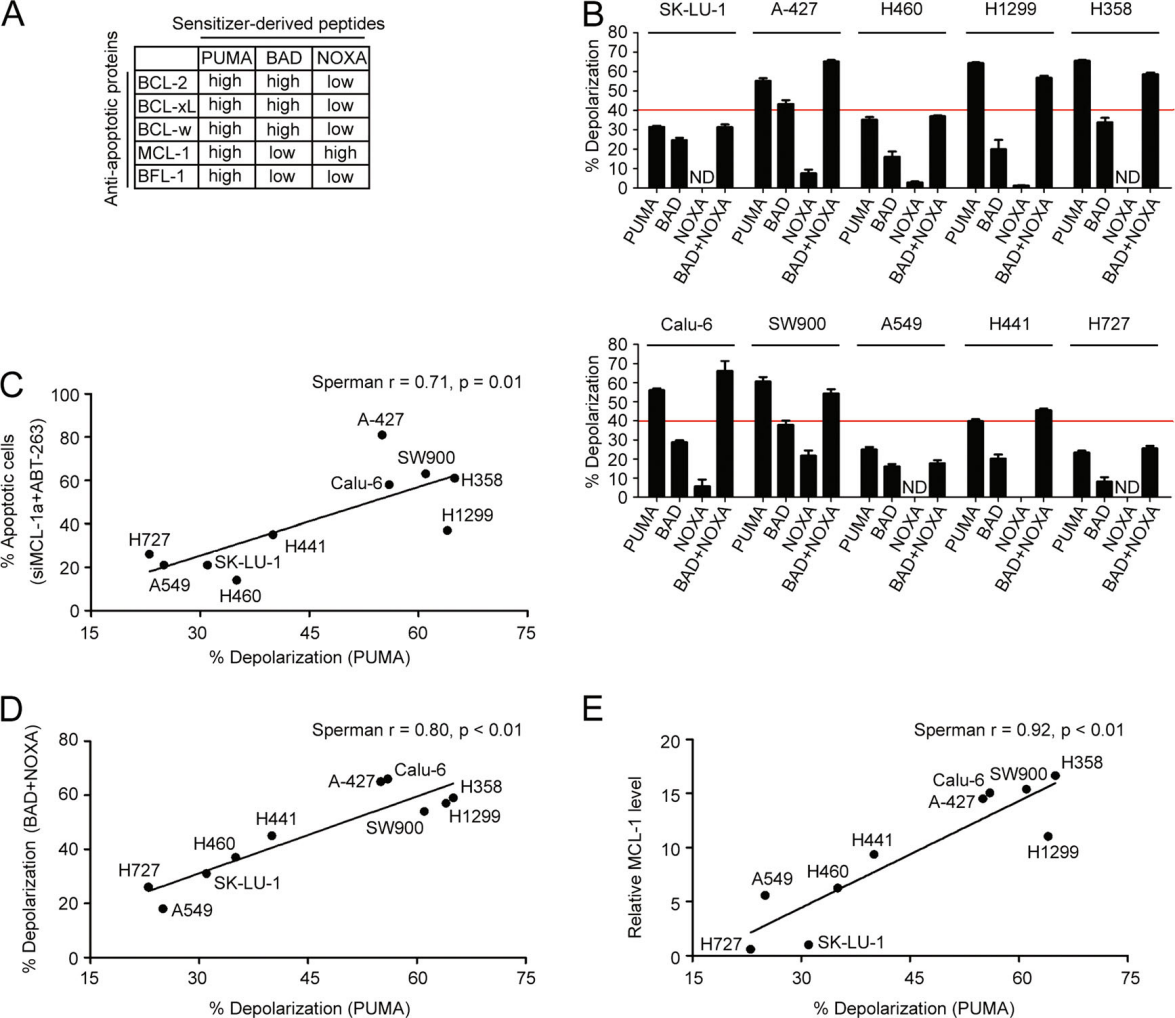
Sensitivity to MCL-1 and BCL-xL inhibition positively correlates with mitochondrial priming. **a** Binding affinity of PUMA, BAD, and NOXA-derived peptides to the BCL-2 family proteins. **b** BH3 profiling of 10 NSCLC cell lines using indicated peptides (60 μM of each peptide). The mean responses induced by the PUMA, BAD, or NOXA-derived peptides are shown. A red line is placed at the 40% level to show degree of sensitivity from each treatment. There were three samples in each group. ND, not detected. **c** Correlation between siMCL-1 and ABT-263 induced apoptosis and the PUMA peptide-induced depolarization. **d** Correlation between BAD and NOXA peptide-induced depolarization and the PUMA peptide-induced depolarization. **e** Correlation between MCL-1 expression level and the PUMA peptide-induced depolarization

It has been shown that the cellular status of the apoptosis machinery can be determined through BH3 profiling, a process that involves incubation of permeabilized tumor cells with BH3-only protein-derived peptides followed by a measurement of the permeability of the mitochondrial outer membrane which is a parameter indicative of cytochrome C leakage and consequently cell apoptosis^12,25^. Since each peptide preferentially binds to a unique set of anti-apoptotic proteins in the mitochondria (Fig. 4a)^26^, this assay can assign the contribution of individual BH3 proteins on cell apoptosis. The PUMA-derived peptide interacts with all five anti-apoptotic proteins (Fig. 4a), and its effect on depolarization reflects the overall cellular response following the inhibition of all anti-apoptotic proteins (Note that although the full-length PUMA protein is an activator, the PUMA-derived peptide fails to activate BAX or BAK directly^24,26,27^). In comparison, results from the BAD and NOXA peptides can only provide partial cellular response from the apoptotic machinery, as the BAD peptide binds to three of the five anti-apoptotic proteins and MCL-1 is the only target for the NOXA peptide (Fig. 4a). Here, the PUMA2A peptide, which is a double alanine-substituted PUMA peptide, was set as a negative control and it did not affect the cell depolarization at concentrations as high as 120 μM (Fig. S4A and B). Applying this approach, we probed all 10 NSCLC cells with 60 μM of peptides derived from PUMA, BAD, and NOXA, and measured the percentage of depolarization as a readout for mitochondrial outer membrane permeability. Interestingly, there were two groups of cells that showed a distinct response pattern to the PUMA-derived peptide treatment. Cells that responded well to the ABT-263 and siMCL-1 combination treatment also displayed a high percentage of depolarization upon treatment with the PUMA peptide (Fig. 4b, c). In line with the expectation that both BCL-xL and MCL-1 played critical roles in mediating anti-apoptosis, BAD and NOXA single treatment did not affect depolarization in any of these cell lines, and the combined effect matched with that from PUMA (Fig. 4b, d). Note that the effect of the combination of BAD and NOXA cannot be explained by the increased final total peptide concentration (120 μM) since the increase of the concentration of BAD or NOXA-derived peptides from 60–120 μM did not show a significant enhancement of cell depolarization (Fig. S4A and B).

There was also a correlation between MCL-1 expression and PUMA peptide-induced depolarization (Fig. 4e). This raises the possibility that MCL-1 expression has to reach a threshold in NSCLC cells in order for the apoptosis machinery to function properly. To test this hypothesis, we knocked down MCL-1 expression in H358 and Calu-6, the high MCL-1 expression cell lines, and overexpressed MCL-1 in H727 and A549, the low MCL-1 expression cell lines (Fig. S5A, C, E, and G). BH3 profiling analysis revealed that, although the PUMA peptide-induced response did not change in H358 and Calu-6 cells, the BAD peptide-induced response increased significantly following MCL-1 knockdown (Fig. S5B and D). On the other hand, MCL-1 overexpression in H727 and A549 cells did not change the percentage of depolarization at all (Fig. S5F and H). The results indicate that, while MCL-1 is a key factor, mitochondrial priming potential can also be affected by additional proteins inside the tumor cells.

### Docetaxel enhances mitochondrial priming and sensitizes NSCLC cells to BCL-xL inhibition

**Fig. 5:**
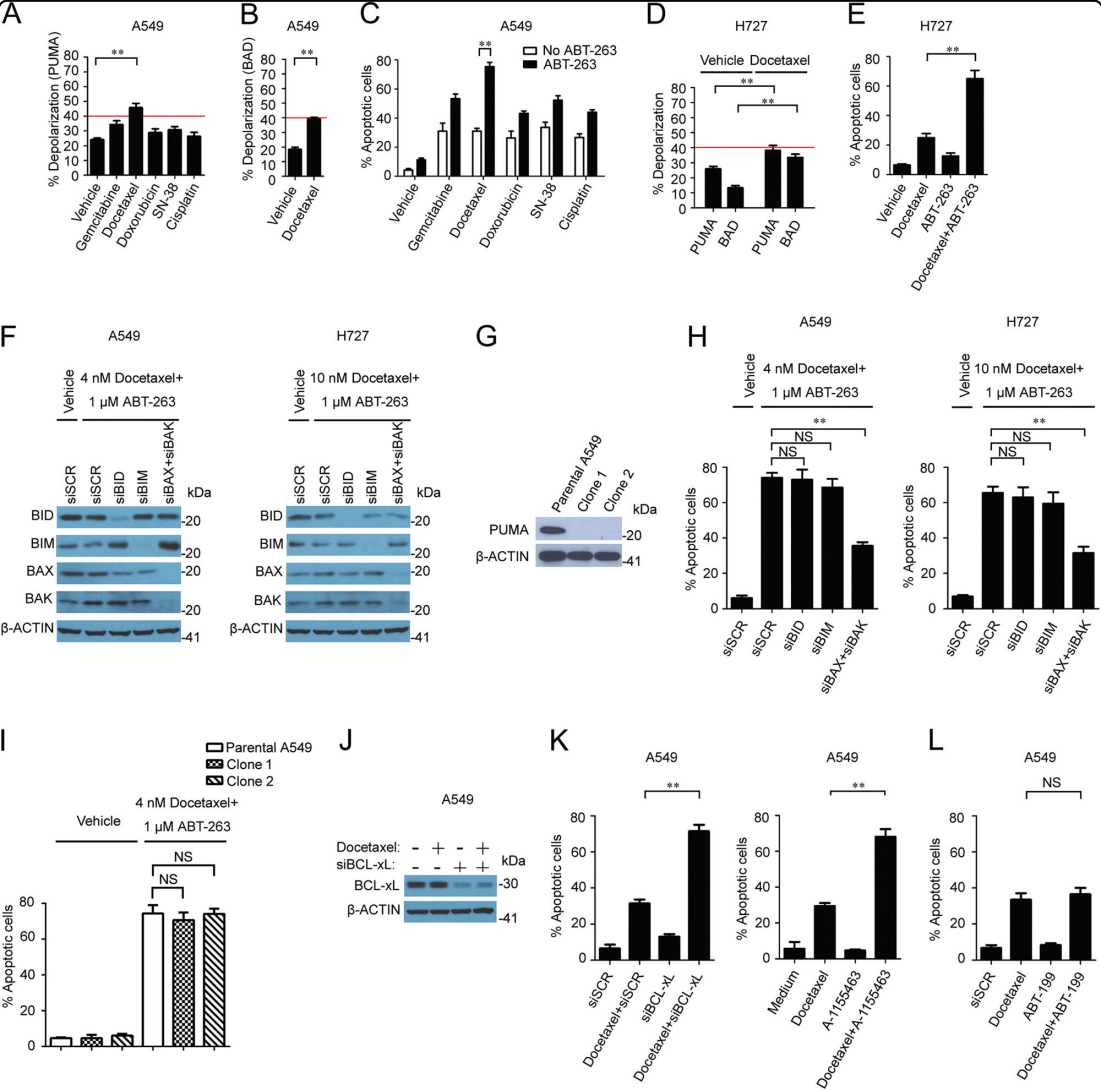
Docetaxel sensitizes low MCL-1-expressing cells to BCL-xL inhibition by enhancing mitochondrial priming. **a** 60 μM of PUMA peptide-induced depolarization in A549 cells treated with chemotherapy drugs at their IC50 levels. A red line is placed at the 40% level to show degree of sensitivity from each treatment. **b** 60 μM of BAD peptide-induced depolarization in A549 cells treated with 4 nM docetaxel. **c** Docetaxel sensitizes A549 cells to ABT-263 treatment. A549 cells were treated with indicated chemotherapy drugs at their IC50 levels +/- ABT-263 for 72 h and flow cytometry was applied to measure cell apoptosis. There were three samples in each group. **d** Docetaxel enhances sensitivity of H727 cells to PUMA and BAD peptide-induced responses. H727 cells were treated with 10 nM docetaxel for 72 h and PUMA and BAD-induced responses were measured by BH3 profiling. A red line is placed at the 40% level to show degree of sensitivity from each treatment. **e** Docetaxel synergizes with ABT-263 on H727 cell apoptosis. H727 cells were treated with 10 nM docetaxel +/- ABT-263 for 72 h and flow cytometry was performed to measure cell apoptosis. There were three samples in each group. **f** Expression analysis in A549 and H727 cells after treatment with 25 nM siRNA oligos for 48 h followed by the indicated drugs for another 24 h. **g** Western blot analysis on PUMA expression in parental and CRISPR-Cas9 engineered A549 cells. **h** Cell apoptosis analysis in A549 and H727 cells treated with 25 nM siRNA oligos followed by docetaxel and ABT-263 for 72 h. There were three samples in each group. **i** Cell apoptosis analysis in the parental A549 and CRISPR-Cas9 engineered A549 cells treated with docetaxel and ABT-263 for 72 h. There were three samples in each group. **j** Expression analysis in A549 cells treated with 25 nM siRNA for 48 h followed by treatment with 4 nM docetaxel for another 24 h. **k** Synergistic killing of A549 cells from docetaxel combined with siBCL-xL or A-155463. A549 cells were treated with 25 nM siRNA for 48 h followed by 4 nM docetaxel treatment for 72 h (left). A549 cells were treated with 4 nM docetaxel and 100 nM A-1155463 for 72 h (right). There were three samples in each group. **l** No synergistic killing of A549 cells from docetaxel and ABT-199 combination treatment. A549 cells were treated with 4 nM docetaxel and 1 μM ABT-199 for 72 h. There were three samples in each group

Since most chemotherapy drugs kill cancer cells by triggering cell apoptosis, we applied them to A549 cells in order to test if the effect is related to an enhancement of the mitochondrial priming potential. At the IC50 level (Fig. S6A-E), docetaxel was more effective in promoting PUMA peptide-induced response than other common chemotherapy drugs for NSCLC (Fig. 5a). This drug also raised BAD peptide-induced response in A549 cells (Fig. 5b). Consistently, co-incubation of docetaxel with ABT-263 stimulated the strongest apoptotic effect compared to the other chemotherapy drugs (Fig. 5c). The effect was not limited to A549 cells, as H727 cells treated with docetaxel at the IC50 concentration (Fig. S6F) also showed a significantly increased percentage of cellular depolarization upon treatment with PUMA or BAD-derived peptide (Fig. 5d). In line with the BH3 profiling results, enhanced cell killing by docetaxel was observed when combined with ABT-263 in H727 (Fig. 5e). Using Compusyn software, the extent of the interaction between docetaxel and ABT-263 in A549 and H727 was investigated. As shown in Table 2, very strong synergisms were observed by simultaneous exposure of A549 to docetaxel (2.22–60 nM) and ABT-263 (0.22–6 µM), and moderate to very strong synergisms were obtained in H727 by simultaneous exposure to docetaxel (6.67–60 nM) and ABT-263 (0.67–6 µM).

**Table 2:** Combination index values for combined treatment of docetaxel and ABT-263 in A549 and H727

Docetaxel (nM)	ABT-263 (µM)	A549			H727		
		Viable cell (%)	CI	Degree of synergism/antagonism	Viable cell (%)	CI	Degree of synergy/antagonism
60.00	6.00	1.2 ± 0.2	0.004	Very strong synergistic	2.4 ± 0.2	0.118	Strong synergistic
20.00	2.00	1.4 ± 0.4	0.001	Very strong synergistic	6.5 ± 1.7	0.092	Very strong synergistic
6.67	0.67	13.4 ± 1.8	0.010	Very strong synergistic	49.1 ± 2.8	0.356	Moderate synergistic
2.22	0.22	32.8 ± 2.1	0.069	Very strong synergistic	75.7 ± 2.5	0.445	Moderate synergistic
0.74	0.07	57.5 ± 1.9	0.805	Moderate synergistic	94.5 ± 3.4	1.225	Moderate antagonistic

A549 or H727 cells were treated with different concentrations of docetaxel, ABT-263 and their respective combinations as indicated. The percentage of viable cell numbers was quantified by MTT assay. CI values were calculated using the CompuSyn, Inc. software program

To identify factors that were involved in docetaxel-mediated cell killing, we suppressed the expression of the BH3-only activators (BIM, BID, and PUMA) and effector proteins (BAX and BAK) in the apoptotic machinery (Fig. 5f, g), and measured apoptotic responses following a combination treatment with ABT-263 and docetaxel. Knockdown of either BIM or BID did not alter the percentage of apoptotic cells (Fig. 5h). PUMA has been reported as a critical mediator of the apoptotic responses induced by agents that cause DNA damage^28^, but the knockout of PUMA did not protect A549 from the combination treatment either (Fig. 5i), indicating that none of these BH3-only activators alone determines the response of the combination treatment. In comparison, knockdown of BAX and BAK significantly rescued cells (Fig. 5h), indicating that the cell killing effect from the ABT-263 and docetaxel combination relies on the release of cytochrome C and other pro-apoptotic factors from the mitochondria via the effector proteins.

To examine whether BCL-xL was also a critical anti-apoptotic protein in the non-responsive lines, we inhibited the function of BCL-xL in A549 cells by either RNA interference (Fig. 5j) or a specific inhibitor, and treated cells with docetaxel. Massive cell apoptosis was observed in cells treated with docetaxel combined with either BCL-xL siRNA or BCL-xL-specific inhibitor S63845 (Fig. 5k). In a separate study, we treated A549 cells with docetaxel, ABT-199, or their combination, and measured cell apoptosis. No synergy was observed from the combination treatment (Fig. 5l). The results indicate that, as in the responsive NSCLC cell lines, BCL-xL played a key role in mediating anti-apoptosis in the non-responsive lines.

### Deletion of MCL-1 expression sensitizes NSCLC tumors to ABT-263 treatment

**Fig. 6:**
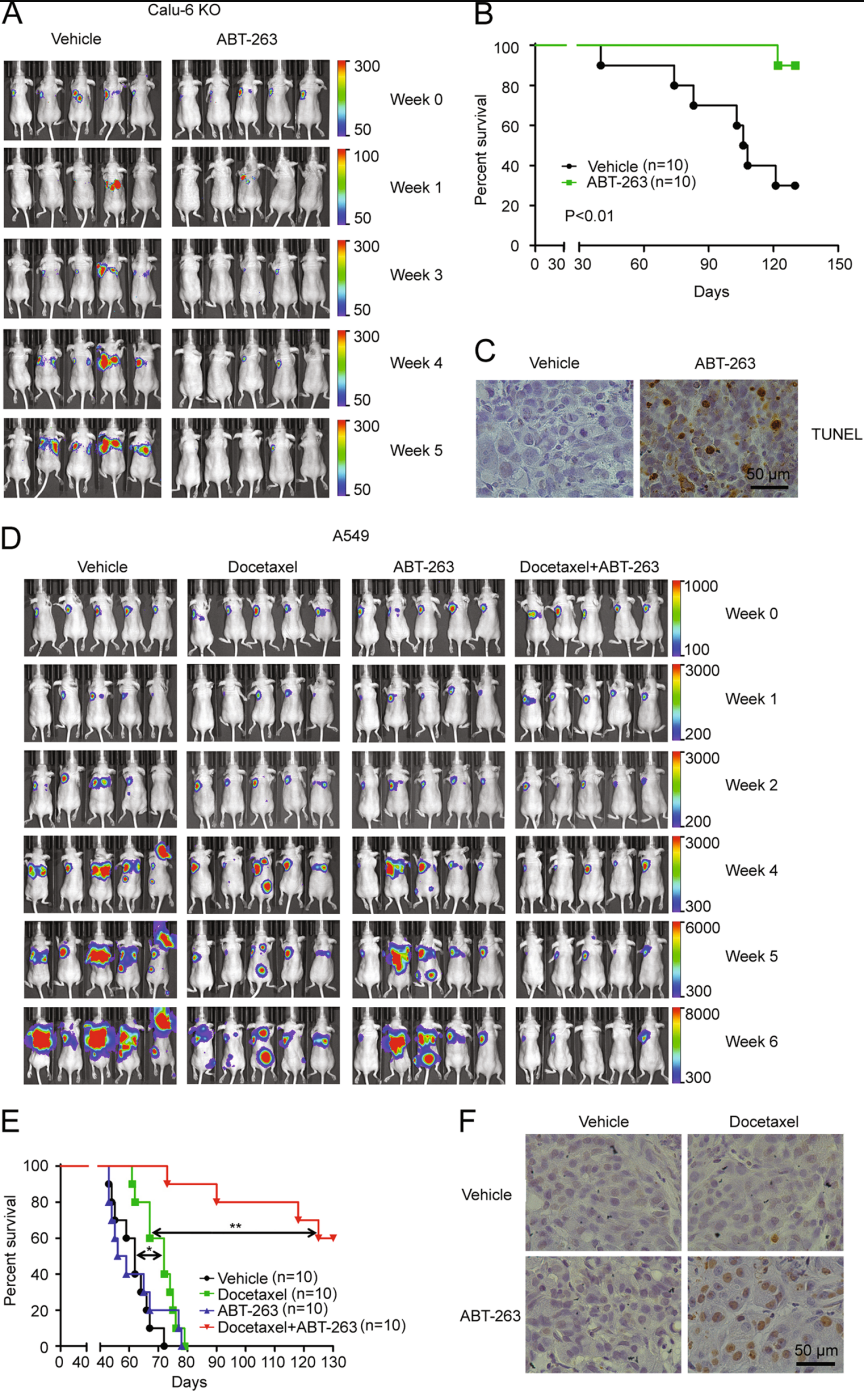
ABT-263 sensitizes NSCLCs to treatment in orthotopic lung tumor models. **a** Athymic nude mice with luciferase-expressing Calu-6 KO tumors were treated with the vehicle control or ABT-263, and tumor growth was monitored with an IVIS imaging system. Representative images from five mice per group are shown. **b** Survival curves of mice with Calu-6 KO tumor after treatment with the vehicle control or ABT-263. Statistical analysis was performed with the Gehan-Breslow-Wilcox test. **c** Representative TUNEL staining images of Calu-6 KO tumors in the vehicle control and ABT-263 treatment groups. **d** Athymic nude mice with A549 tumors were treated with the indicated agents and imaged for tumor growth using an IVIS imaging system. Representative images from five mice per group were shown. **e** Survival curves of mice with A549 tumors following different treatments. Statistical analysis was performed with the Gehan-Breslow-Wilcox test (*P < 0.05, **P < 0.01). **f** Representative TUNEL staining results of A549 tumor from each treatment group

We performed an in vivo efficacy study to validate the observation that NSCLCs with a high MCL-1 expression were sensitive to MCL-1 suppression and ABT-263 combination therapy using a Calu-6 orthotopic lung tumor model. As expected, the Calu-6 tumor did not respond to ABT-263 treatment alone based on tumor growth and animal survival (Fig. S7A-C). In order to eliminate MCL-1 expression in Calu-6 cells, we performed CRISPR-Cas9-based genome editing^29,30^ to delete the MCL-1 gene. Western blot analysis confirmed successful knockout of the MCL-1 gene, as no MCL-1 expression could be detected in the 3 Calu-6 clones (Fig. S7D). In line with the observation that Calu-6 cells were sensitive to MCL-1 siRNA and ABT-263 treatment (Fig. 2c), the MCL-1 knockout clones (Calu-6 KO) were highly sensitive to ABT-263 in a cell-based assay (Fig. S7E). We pooled all of the clones together to generate murine orthotopic tumor models and treated mice with ABT-263. Treatment of the tumor-bearing mice with ABT-263 dramatically inhibited tumor growth and significantly extended animal survival (Fig. 6a, b). Histological analysis of the post-treatment tumor samples revealed a high percentage of apoptotic cells (Fig. 6c).

### Docetaxel and ABT-263 combination treatment significantly extends survival in animals with low MCL-1 expression tumors

Our cell-based study revealed that BCL-xL contributed to chemotherapy resistance in NSCLCs with a low level of MCL-1 (Fig. 5k). To confirm this observation, we treated mice bearing A549 primary tumors with docetaxel, ABT-263, or a combination of the two, and monitored therapeutic responses. As expected, ABT-263 treatment alone had no effect on tumor growth (Fig. 6d). Treatment with docetaxel extended animal survival by 10 days (*P* < 0.05). In comparison, combination of ABT-263 and docetaxel dramatically promoted tumor regression and significantly extended survival over docetaxel treatment alone (*P* < 0.01, Fig. 6e). TUNEL staining revealed massive apoptosis in tumor tissues but not in normal tissues following the combination treatment (Fig. 6f and Fig. S8A, B), suggesting this combination treatment may be effective and tolerable in vivo.

## Discussion

Since preclinical and clinical studies with small molecule inhibitors of the BCL-2 family proteins have shown promising results in a number of cancer types, we evaluated the potential of these inhibitors on NSCLC.

While the small molecule inhibitor targeting BCL-2/BCL-xL/BCL-w (ABT-263) as a single agent has entered clinical experimentation in certain cancers, NSCLC is largely resistant to these inhibitors alone (Fig. S2A). MCL-1 is another critical regulator of cell death in NSCLC^31,32^. Here, we show that all cell lines used in our study did not respond to the knockdown of MCL-1. By contrast, Zhang et al. have observed potent cell killing in four NSCLC cell lines (H23, H1568, H522, and H838) after the inhibition of MCL-1 alone, suggesting there is a subgroup of NSCLC cell lines that is mainly dependent on MCL-1 for survival^10^. Indeed, a recent report investigated the role of MCL-1 in 20 NSCLC cell lines using a potent MCL-1 inhibitor and showed that only 5% of NSCLC cell lines, in contrast with 68% of multiple myeloma cell lines, are highly sensitive to MCL-1 inhibition^33^. Based on this data and our observations, we speculated that most, if not all, of NSCLC cell lines are resistant to MCL-1 inhibition alone.

Although there is little cell death with the inhibition of a single BCL-2 anti-apoptotic protein, suppression both of MCL-1 and BCL-2/BCL-xL/BCL-w induced extensive apoptosis in a subgroup of NSCLC cell lines. A previous study has shown that inhibiting the function of MCL-1 in H1299 by exogenous expression of NOXA sensitized cells to ABT-737, suggesting that the MCL-1 expression level determines the resistance to the inhibition of BCL-2/BCL-xL/BCL-w in NSCLC cells^32^. However, this proposal has not been validated across a panel of cells and cannot explain why a subpopulation of NSCLC cells with a low level of MCL-1 such as A549 are also resistant to BCL-2/BCL-xL/BCL-w inhibition (Fig. 2a and S2A). In the present study, we systematically evaluated the relationship between apoptotic responses after the treatment of MCL-1 antisense oligonucleotide in combination with ABT-263 and levels of BCL-2 family proteins in ten cell lines, results showed only the level of MCL-1 could be a useful biomarker to predict the extent of apoptosis induced by the combination treatment. BH3 profiling demonstrated that cells expressing a low level of MCL-1 were not sensitive to the inhibition of anti-apoptotic proteins because of their low mitochondrial priming.

Previous studies have reported that ABT-263 or ABT-737 enhanced the antitumor activity of chemotherapy regimens^8,34^. Here, in the MCL-1 low-expressing cell line A549, we performed a combination drug screen using ABT-263 with the five most widely used chemotherapeutic agents in NSCLC. The results showed that docetaxel combined with ABT-263 triggered the most potent apoptosis. Notably, the ratio of BCL-xL to MCL-1 has previously been shown to be a biomarker to predict the extent of synergy between taxanes and ABT-263 in NSCLC^8^. Considering that the levels of BCL-xL only varied by around 2.2-fold whereas the levels of MCL-1 varied by around 20-fold in the cell lines used in our study (Fig. 2a and Fig. 3a), the ratio of BCL-xL to MCL-1 is mainly determined by the levels of MCL-1. Indeed, although the ratio of BCL-xL to MCL-1 has a better correlation, this report also showed that the MCL-1 protein level is reversely correlated with response of the combination treatment by an assay performed in 25 cell lines. Thus, it is rational for us to treat cells with a low level of MCL-1, which were resistant to inhibition of BCL-xL and MCL-1, with the combination of docetaxel and ABT-263.

In conclusion, we have identified two groups of NSCLC cells based on their MCL-1 expression levels and developed strategies to effectively treat them by inducing cell apoptosis. For every NSCLC patient, treatment design should be based on molecular profiling especially the MCL-1 expression level. Patients with high MCL-1-expressing NSCLCs should be treated with BCL-xL and MCL-1-targeted therapy, while the chemotherapy should be applied in patients with a low MCL-1 expression in order to sensitize BCL-xL-targeted therapy.

## Electronic supplementary material

Supplement figure legends

Figure S1

Figure S2

Figure S3

Figure S4

Figure S5

Figure S6

Figure S7

Figure S8
