# Supplementary material for: Sensitizing non-small cell lung cancer to BCL-xL-targeted apoptosis
Source: Cell Death Dis. 2018 Sep 24;9(10):986. doi: 10.1038/s41419-018-1040-9 (PMC6155218; doi:10.1038/s41419-018-1040-9)
Supplement: Supplementary file 1 — Supplement figure legends [file 41419_2018_1040_MOESM1_ESM.docx]

**Supplement figure legends**

Figure. S1. Survival of NSCLC patients with anti-apoptotic protein amplification. (A) Survival of NSCLC patients with or without MCL-1 amplification. (B) Survival of NSCLC patients with or without BCL-xL amplification. P-values in A and B were calculated using Gehan-Breslow-Wilcox test. wt, wild type; amp, amplification. (C) Validation of MCL-1 and BCL-xL antibodies by western blot analysis in A549 cells. 20 µg of A549 whole-cell lysate was subjected to SDS-PAGE followed by western blot analysis using antibodies as indicated. No non-specific band can be observed by western blot analysis with the antibody to MCL-1 or BCL-xL. (D) Representative immunohistochemical staining results of adjacent normal lung tissue. NA, not available.

Figure. S2. Differential response from NSCLC cells to combinational treatment of MCL-1 siRNA and ABT-263. (A) Flow cytometry analysis on cell apoptosis upon treatment with siMCL-1a and ABT-263. Cells were treated with siRNA for 48 hours followed by ABT-263 for 36 hours. (B) Dose-response curves of cells to ABT-263 in the presence or absence of MCL-1 knockdown. Cells were treated with siRNA for 48 hours followed by different doses of ABT-263 for 36 hours. Cell viability was analyzed with the MTT assay. All samples were in triplicate and each treatment was repeated 3 times. Representative plots from one experiment are shown. The IC50 value for ABT-263 was calculated with a sigmoid dose-response model using the Graph Pad Prism Software 5.01. (C) The sensitive cell lines have significantly smaller IC50 values than the resistant cell lines (*P < 0.05). (D) Knockdown of MCL-1 expression in H358 cells with a second siMCL-1. H358 cells were transfected with 25 nM siMCL-1s for 48 hours before they were harvested for expression analysis. (E) Flow cytometry analysis on cell apoptosis upon treatment with siMCL-1b and ABT-737. H358 cells were treated with siRNA for 48 hours followed by ABT-737 for 36 hours. (F) Knockdown of MCL-1 and BCL-xL expression in NSCLC cells after transfection with 25 nM siRNA oligos. Cells were treated with 25 nM siRNA for 48 hours before they were harvested for expression analysis.

Figure. S3. MCL-1 siRNA and ABT-263 combinational treatment selectively targets a subset of NSCLC cells. (A) Knockdown of MCL-1 expression in a normal lung cell line MCR-5. Cells were treated with 25 nM siRNA for 48 hours followed by 1 μM ABT-263 for 36 hours. (B) siMCL-1 and ABT-263 does not trigger apoptosis in MCR-5 normal lung cells. MCR-5 cells were treated with 25 nM siRNA for 48 hours followed by 1 μM ABT-263 for 36 hours before being analyzed for cell apoptosis. (C) MCL-1 small molecule inhibitor S63845 synergizes with ABT-263 in triggering cell apoptosis. Cells were treated with 500 nM S63845 and 1 μM ABT-263 for 48 hours before being analyzed for cell apoptosis. (D) Significant correlation between siMCL-1a-induced and S63845-induced cell apoptosis when each are combined with ABT-263.

Figure. S4. Increasing the concentration of PUMA2A, BAD, or NOXA-derived peptides does not enhance the depolarization in cells. H358 cells (A) and A549 cells (B) were analyzed by BH3 profiling using 60 μM or 120 μM of indicated peptides. There were 3 samples in each group. NS: P>0.05. ND, not detected.

Figure. S5. Modulation of cell apoptosis by MCL-1 expression. (A-D) Knockdown of MCL-1 in H358 cells or Calu-6 cells sensitizes cells to BAD-specific peptide. H358 cells or Calu-6 cells were transfected with 25 nM siRNA for 48 hours and cells were analyzed for protein expression (A and C) and BH3 profiling using indicated peptides (60 μM of each peptide) (B and D). A red line is placed at the 40% level to show degree of sensitivity from each treatment. NS: P>0.05; *: p<0.05; **: p<0.01. ND, not detected. (E-H) MCL-1 overexpression does not enhance sensitivity in H727 cells or A549 cells. H727 cells or A549 cells were transfected with either the PCMV6-XL4 expression vector or the MCL-1 overexpression plasmid. MCL expression (E and G) and BH3 profiling using indicated peptides (60 μM of each peptide) (F and H) were examined 48 hours later. ND, not detected. A red line is placed at the 40% level to show degree of sensitivity from each treatment.

Figure. S6. Dose-response curves of A549 cells and H727 cells to chemotherapy drugs. (A-E) A549 cells were incubated with different doses of gemcitabine, docetaxel, doxorubicin, SN-38, or cisplatin for 72 hours. Cell survival was analyzed with the MTT assay. (F) H727 cells were incubated with increasing concentrations of docetaxel for 72 hours. Cell viability was analyzed with the MTT assay. All samples were in triplicate and each treatment was repeated 3 times. Representative plots from one experiment are shown. The IC50 value for each drug was calculated with a sigmoid dose-response model using the Graph Pad Prism Software 5.01.

Figure. S7. MCL-1 expression in Calu-6 tumors determines sensitivity to ABT-263 treatment. (A) Mice with Calu-6 tumors were treated with the vehicle control or ABT-263, and imaged for tumor growth with an IVIS imaging system. Representative images from 5 mice per group were shown. (B) Survival curves of mice with Calu-6 tumors after treatment with the vehicle or ABT-263. Statistical analysis was performed with the Gehan-Breslow-Wilcox test (n=10). (C) Representative TUNEL staining images of Calu-6 tumor samples after vehicle or ABT-263 treatment. (D) Western blot analysis on MCL-1 expression in parental and CRISPR-Cas9 engineered Calu-6 cells. (E) Percentage of apoptotic cells after ABT-263 treatment. Calu-6 KO cells were treated with 1 μM ABT-263 for 36 hours. Cell apoptosis was measured using flow cytometry.

Figure. S8. Docetaxel and ABT-263 combinational treatment selectively targets the tumor cells in athymic nude mice with A549 tumors. (A) A representative TUNEL staining image of the normal lung tissue after the combination of docetaxel and ABT-263 treatment. (B) Percentage of TUNEL-positive cells from tumor tissues and from normal lung tissues following the combination of docetaxel and ABT-263 treatment (**P < 0.01).
