# Supplementary figures and images for: Sensitizing non-small cell lung cancer to BCL-xL-targeted apoptosis
Source: Cell Death Dis. 2018 Sep 24;9(10):986. doi: 10.1038/s41419-018-1040-9 (PMC6155218; doi:10.1038/s41419-018-1040-9)

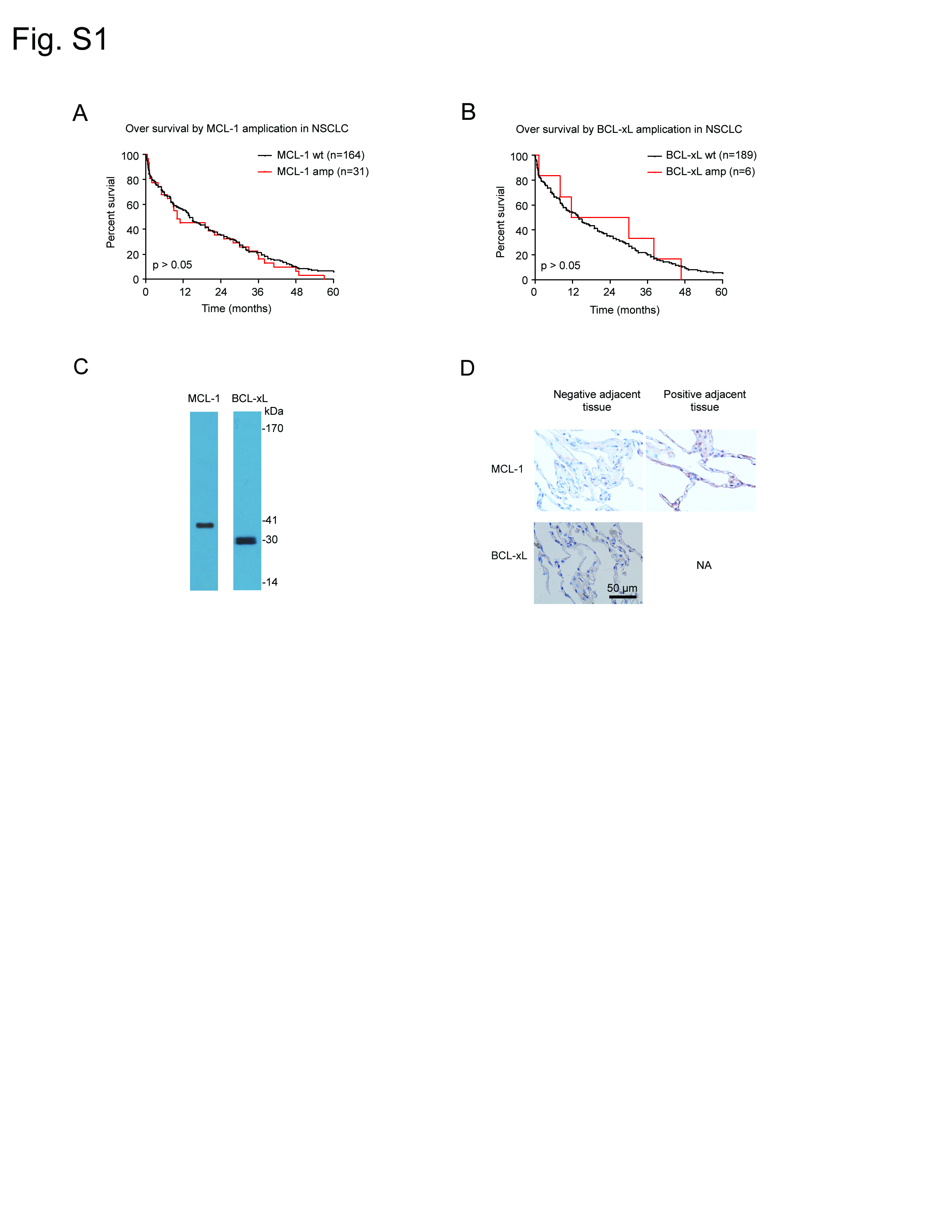

Supplement: Supplementary file 2 — Figure S1 [file 41419_2018_1040_MOESM2_ESM.jpg]

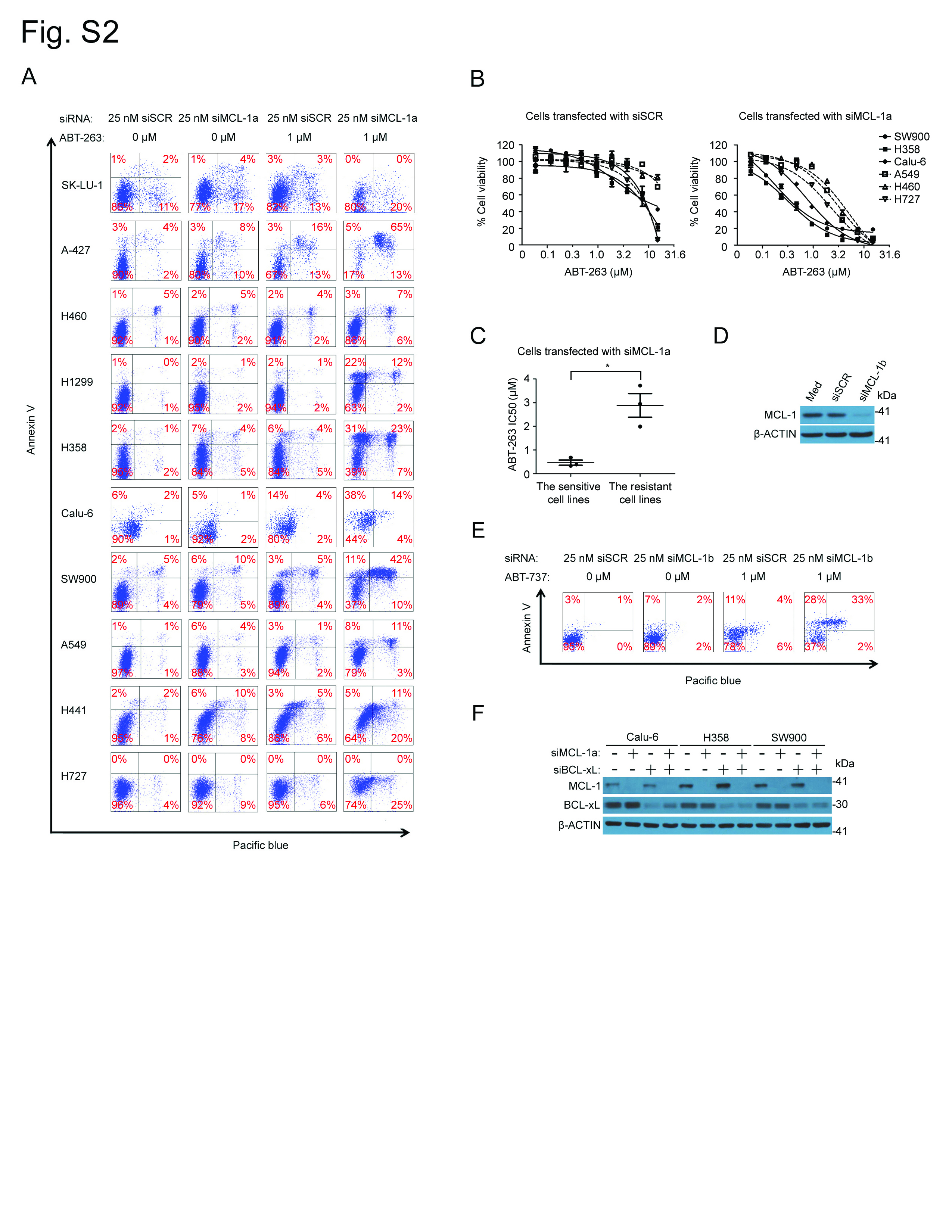

Supplement: Supplementary file 3 — Figure S2 [file 41419_2018_1040_MOESM3_ESM.jpg]

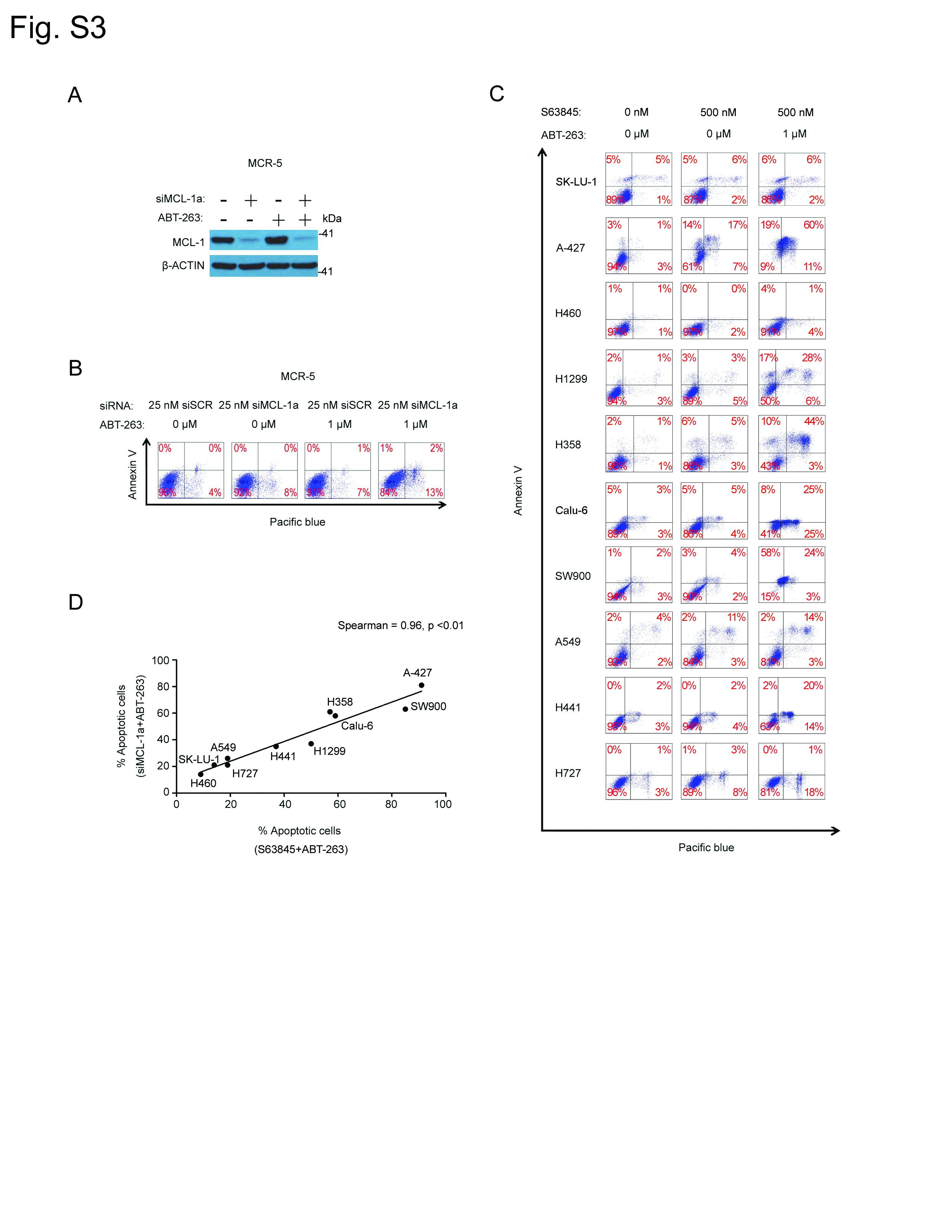

Supplement: Supplementary file 4 — Figure S3 [file 41419_2018_1040_MOESM4_ESM.jpg]

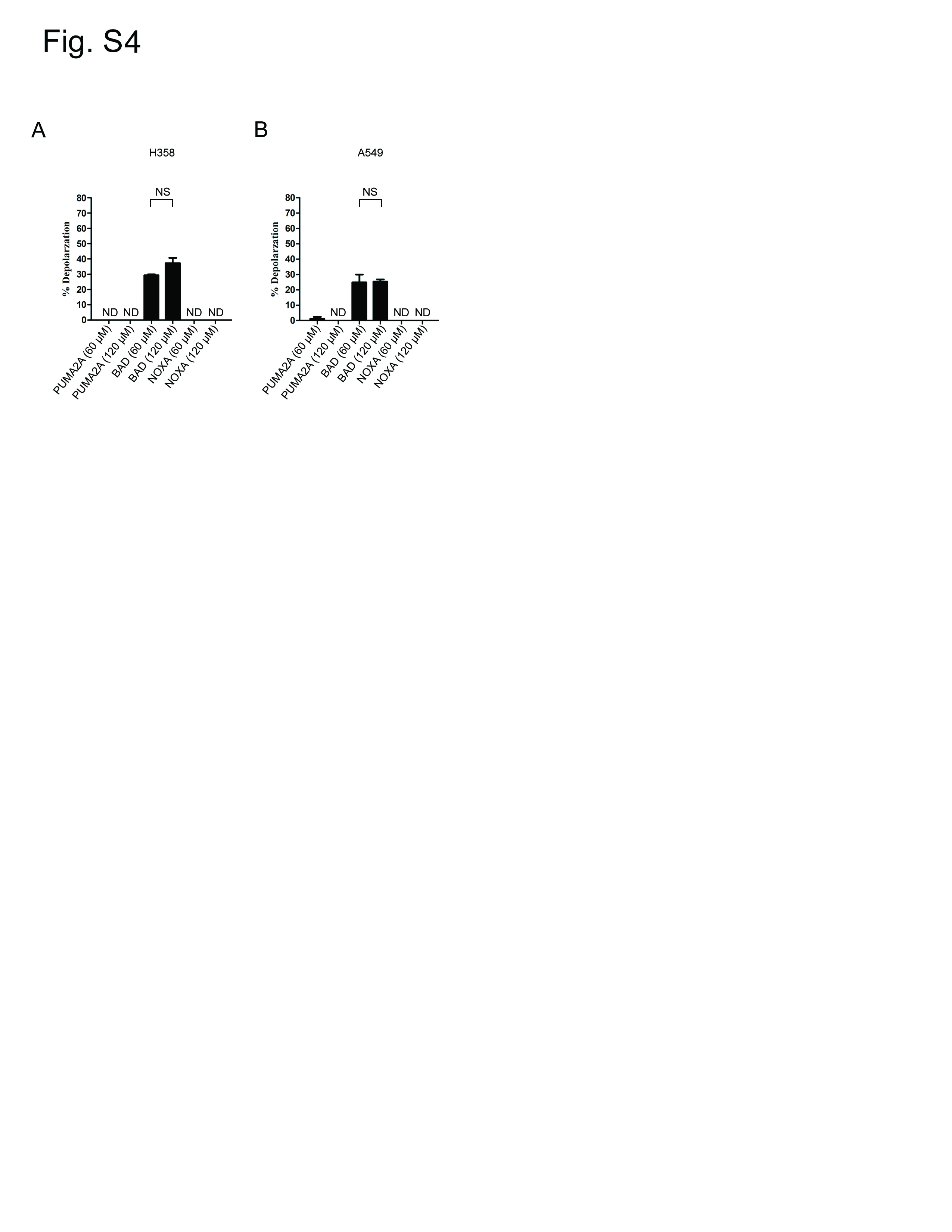

Supplement: Supplementary file 5 — Figure S4 [file 41419_2018_1040_MOESM5_ESM.jpg]

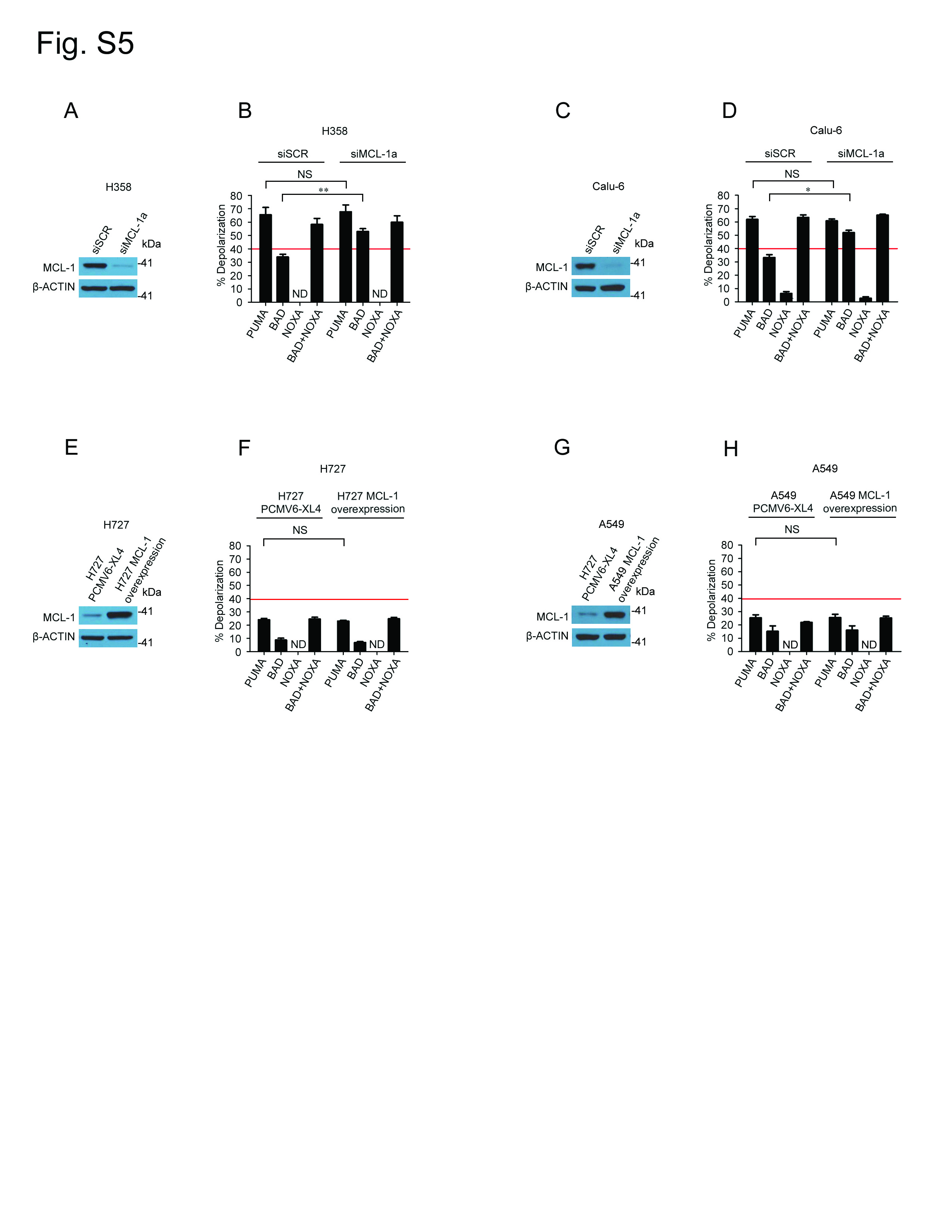

Supplement: Supplementary file 6 — Figure S5 [file 41419_2018_1040_MOESM6_ESM.jpg]

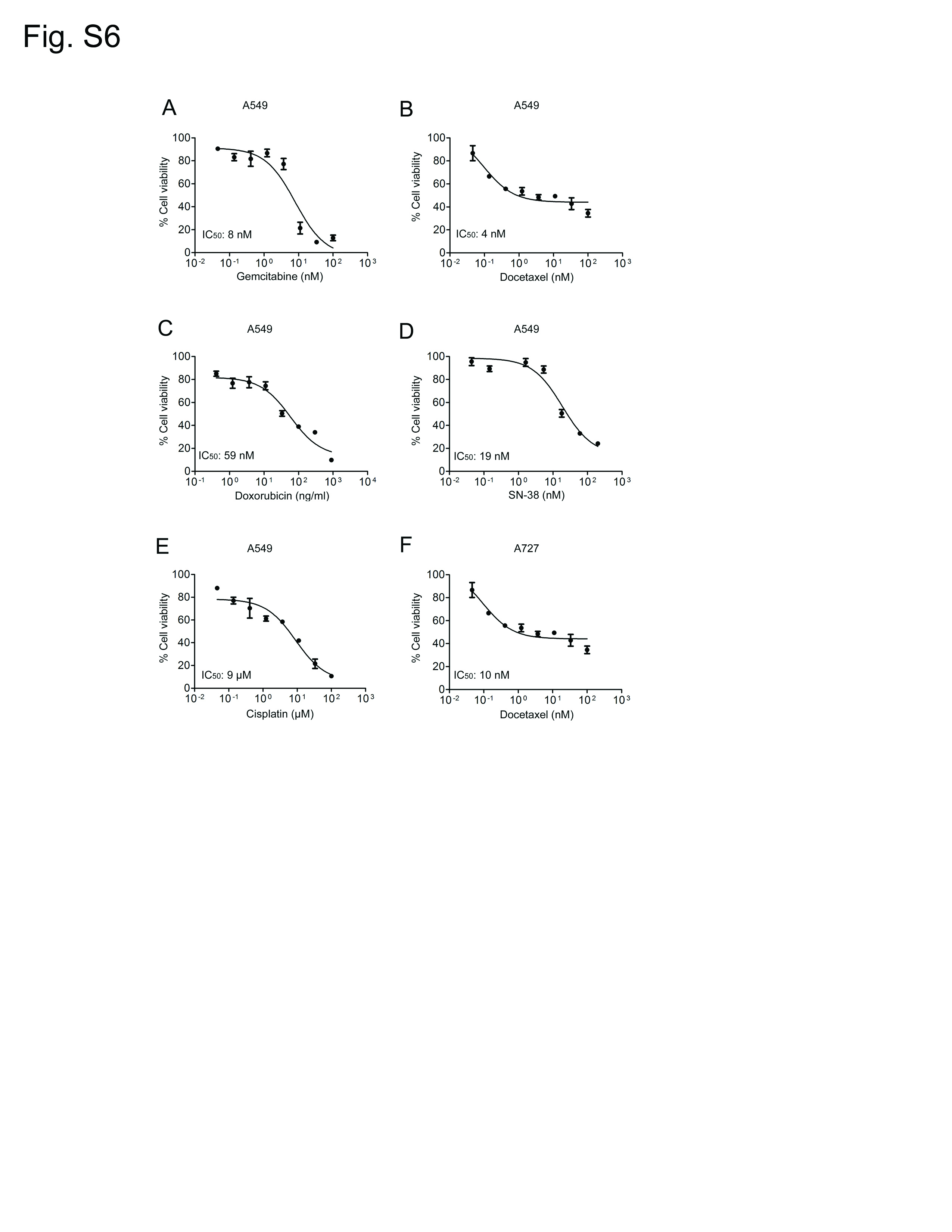

Supplement: Supplementary file 7 — Figure S6 [file 41419_2018_1040_MOESM7_ESM.jpg]

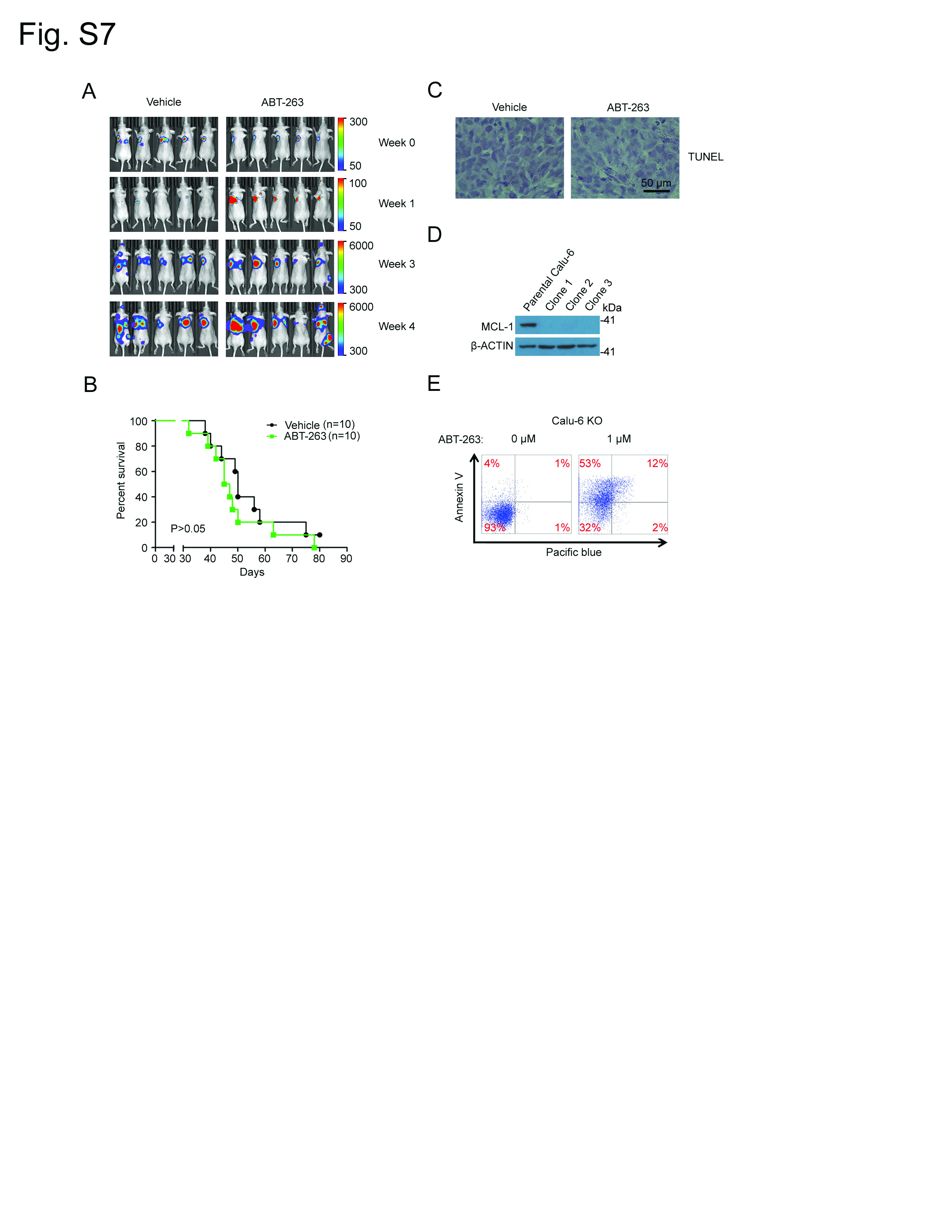

Supplement: Supplementary file 8 — Figure S7 [file 41419_2018_1040_MOESM8_ESM.jpg]

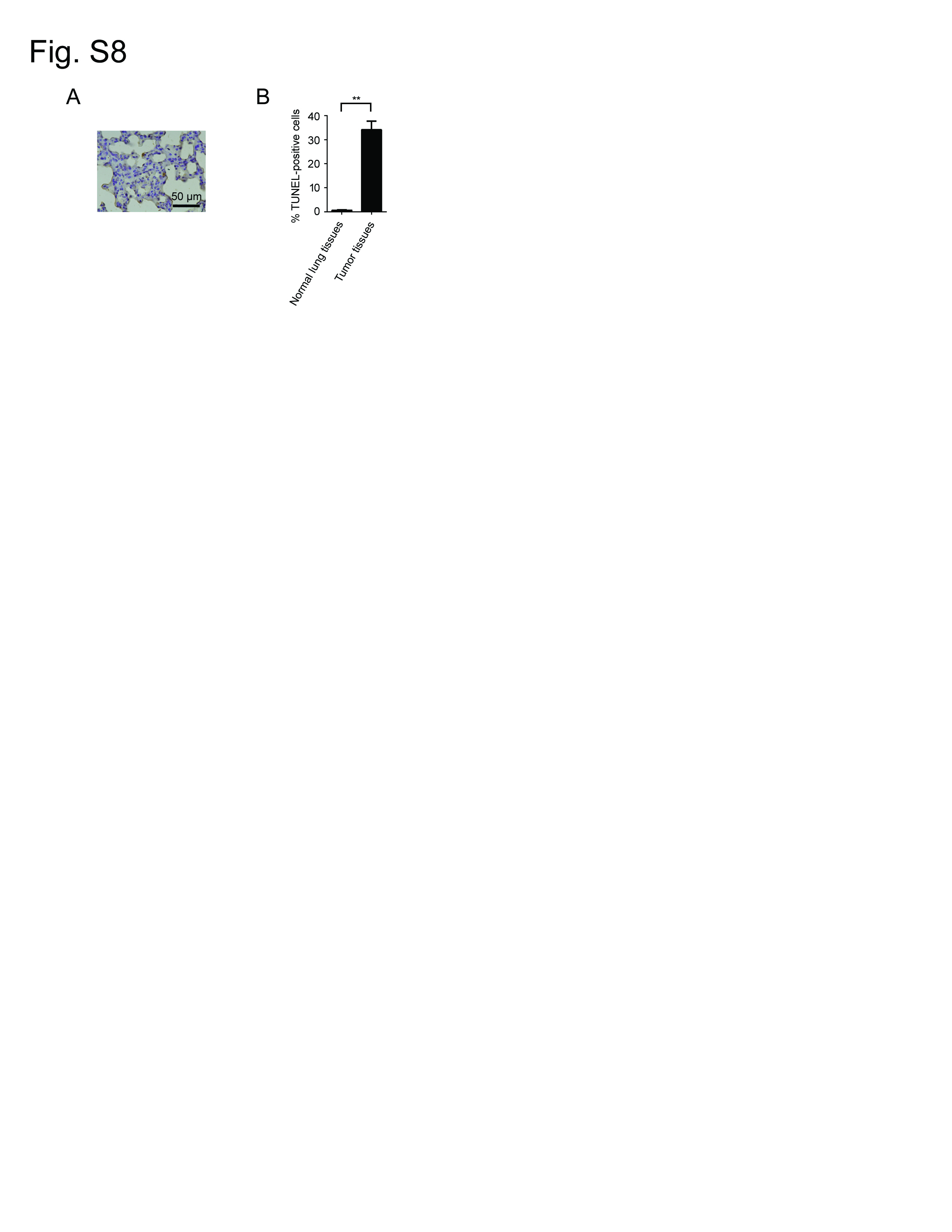

Supplement: Supplementary file 9 — Figure S8 [file 41419_2018_1040_MOESM9_ESM.jpg]
